# TRIM29 knockdown prevented the colon cancer progression through decreasing the ubiquitination levels of KRT5

**DOI:** 10.1515/biol-2022-0711

**Published:** 2023-08-30

**Authors:** Lihui Sun, Dawei Wang, Zhenyu Chen, Xu Zhu

**Affiliations:** The Fifth Department of General Surgery, The Third Affiliated Hospital of Jinzhou Medical University, No. 2, Section 5, Heping Road, Jinzhou, Liaoning 121000, China; The Second Department of General Surgery, Dalian Fifth People’s Hospital, Dalian, Liaoning 116081, China; The Fifth Department of General Surgery, The Third Affiliated Hospital of Jinzhou Medical University, Jinzhou, Liaoning 121000, China

**Keywords:** colon cancer, ubiquitination, TRIM29, KRT5

## Abstract

To investigate the specific role of TRIM29 in colon cancer progression, bioinformatic analysis was performed on TRIM29. Colon cancer tissues were collected and colon cancer cells were cultured for further experiments. Cell viability and proliferation were determined using CCK-8, colony formation, and EDU staining assays. The mRNA and protein levels of TRIM29 and KRT5 were determined using quantitative real-time PCR and western blotting, respectively. The interaction between TRIM29 and KRT5 was detected using a co-immunoprecipitation (CO-IP) assay. Cycloheximide treatment was performed to analyse the stability of KRT5. TRIM29 was upregulated in colon cancer tissues and cells. TRIM29 knockdown decreased the cell viability and proliferation and ubiquitination levels of KRT5 and enhanced the protein stability and expression of KRT5. The CO-IP assay confirmed that TRIM29 and KRT5 binded to each other. KRT5 knockdown neutralises the inhibitory effect of sh-TRIM29 on colon cancer cell growth and TRIM29 knockdown prevented the proliferation of colon cancer cells by decreasing ubiquitination of KRT5, which enhanced the protein stability and expression of KRT5 in cancer cells. Thus, targeting TRIM29-mediated ubiquitination levels of KRT5 might be a new direction for colon cancer therapy.

## Graphical abstract

**Figure:**
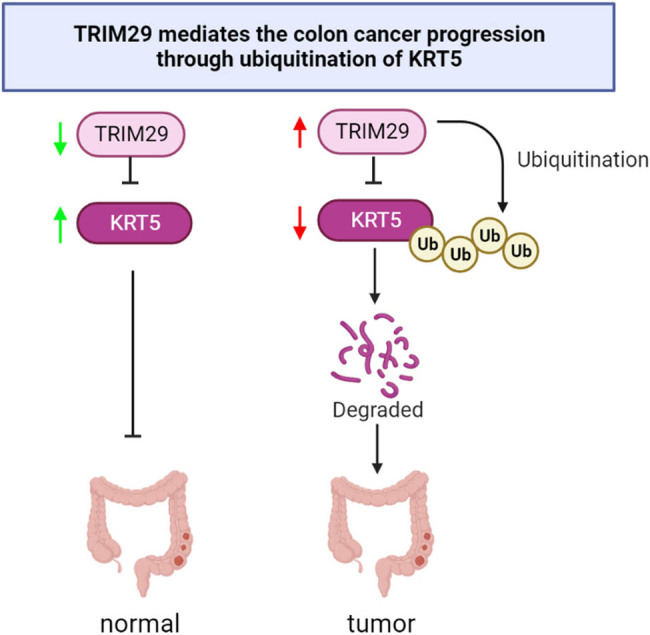

## 1 Introduction

Colon cancer is a common malignant tumour of the digestive system, affecting the junction of the rectum and sigmoid colon. Colon cancer ranks second among the incidence rates of cancer in China [1], and its mortality rate is also high. The average 5-year survival rate of patients with colon cancer is approximately 50%, while the 5-year survival rate of advanced colon cancer is less than 10% [2]. Therefore, it is important to actively identify potential biomarkers of colon cancer for the diagnosis, treatment, and prognosis of colon cancer.

Ubiquitination is an important post-translational modification in eukaryotes that regulates the physiological functions of cells [3]. The ubiquitination-mediated protein degradation pathway plays an important role in cell cycle regulation, cell signal transduction, DNA repair, cell morphology maintenance, protein quality control, and transcriptional regulation [4–6].

Additionally, ubiquitination regulates the stability and function of many oncogenes and tumour suppressor genes. Ubiquitination is catalysed by the ubiquitin E1 activating enzyme, ubiquitin E2 binding enzyme, and ubiquitin E3 ligase [3]. Ubiquitin E3 ligase, as a bridge protein, regulates the interaction between E2 enzyme and its substrate by specifically recognising the substrate [7]. Members of the Triple motif (TRIM) family (also known as the RBCC family) contain a RING-finger domain and thus, function as ubiquitin E3 ligases. Approximately 80 TRIM proteins have been identified in the human genome [8]. TRIM family proteins are involved in many biological processes, and abnormally expressed TRIM family proteins cause a variety of pathological changes such as developmental disorders, neurodegenerative diseases, viral infections, and cancers [9,10]. Most functions of TRIM include E3 ubiquitin ligases involved in tumourigenesis and tumour development by regulating gene expression, cell proliferation, and apoptosis [11].

TRIM containing 29 (TRIM29), also known as the ataxia telangiectasia group D complementing gene (ATDC), is located on 11q23 and is a member of the TRIM family. TRIM29 contains a zinc finger gene sequence and is a transcriptional regulator [12]. TRIM29 is abnormally expressed in many malignant tumours and is involved in the proliferation and metastasis of cancer cells [13,14]. Additionally, many studies have shown that TRIM29 participates in the regulation of malignant behaviours of cancer cells by regulating ubiquitination of target genes such as YAP1 [15], ISG15 [16], and p53 [17]. Keratin (KRT) encodes a group of intermediate filament proteins that constitute the cytoskeleton of epithelial cells [18,19]. Malignant tumour cells often originate from epithelial cells. Previous studies have reported that KRT may play a role in cell apoptosis, cell growth, epithelial polarity, wound healing, and tissue remodelling [20]. Among the KRT family members, KRT5 has been used as a single marker or in combination with KRT6 (KRT5/6) for antigen-specific immunohistochemical diagnosis of squamous cell carcinoma [21–23]. However, the interactions between TRIM29 and KRT5 in the colon remain unclear.

Therefore, this study aimed to investigate the role of TRIM29 in colon cancer progression. We hypothesised that TRIM29 knockdown increases the KRT5 levels and stability by declining the ubiquitination of KRT5.

## 2 Materials and methods

### 2.1 Cancer tissue collection

A total of 26 colon cancer tissue samples were collected as the cancer group and 26 adjacent tissue samples (according to the cutting edge >2 cm) as the normal group, all of which were confirmed via histopathology and frozen at −80°C.


**Informed consent:** Informed consent has been obtained from all individuals included in this study.
**Ethical approval:** The research related to human use has been complied with all the relevant national regulations, institutional policies and in accordance with the tenets of the Helsinki Declaration, and has been approved by hospital ethics committee (No. KX2020007).

### 2.2 Immunohistochemistry

Tumour and normal tissues were embedded in paraffin and cut into 4 µm-thick sections. The sections were de-paraffinised using xylene and rehydrated using a gradient concentration of ethanol; the antigen was extracted using microwaves. Subsequently, the sections were incubated with anti-KRT5 (ab8068; Abcam) at 4°C overnight and then with a secondary antibody (ab288151; Abcam) at room temperature for 30 min. DAB plus substrate (D7679, Sigma-Aldrich) was added and the sections were incubated for 10 min. The results were visualised using a microplate reader.

### 2.3 Analysis of differentially expressed genes in colon cancer

The GSE104836 microarray data for ten tumour tissues and ten normal tissues were downloaded from the GEO database. The GSE104836 dataset was downloaded, pre-processed, and normalised using the GEO query R package. The limma R package was used to screen out differentially expressed genes, and the filter conditions were set as adj.*P* value <0.05 and |log2FC| >2. Differentially expressed genes are expressed as heat and volcano maps.

### 2.4 Cell culture

Normal human colon epithelial cells (NCM460) and colon cancer cell lines (SW480, HCT116, SW620, LoVo, and RKO) were purchased from Wuhan Procell Life Technology Co., Ltd (Wuhan, China). All cells were cultured in DMEM medium containing 10% foetal bovine serum and 1% penicillin streptomycin, and they were placed in a constant temperature incubator at 37°C under 5% CO_2_ and saturated humidity.

### 2.5 Cell transfection

For cell transfection, sh-TRIM29, sh-KRT5, and KRT5, TRIM29 overexpression vectors and their controls (sh-NC and vector) were provided by Shanghai GenePharma Co., Ltd (Shanghai, China) and transfected into cells using LipofectamineTM 2000 (11668019, Invitrogen, USA) according to manufacturer’s instructions. After 48 h, the transfected cells were collected and efficiency was detected using quantitative real-time PCR (qPCR) and western blotting.

### 2.6 qPCR

Total RNA was isolated using the TRNzol Universal reagent (DP424, TianGen, Beijing). Reverse transcription and qPCR were conducted using a one-step RT-QPCR kit (SYBR Green) (FP207-02, TianGen). qPCR was performed on a Line-Gene Real Time PCR system (FQD-A1600, Bioer, Hangzhou, China). The relative expression was analysed using 2^−ΔΔCT^ method. GAPDH was selected as the normalisation. The primer sequences were as follows (5′ → 3′):

TRIM29, Forward Primer, CTGTTCGCGGGCAATGAGT; Reverse Primer, TGCCTTCCATAGAGTCCATGC.

KRT5, Forward Primer, CCAAGGTTGATGCACTGATGG; Reverse Primer, TGTCAGAGACATGCGTCTGC.

GAPDH, Forward Primer, TGTGGGCATCAATGGATTTGG; Reverse Primer, ACACCATGTATTCCGGGTCAAT.

### 2.7 Western blotting

Total proteins were isolated from placental tissues and SW480 and HCT116 cells using radio-immunoprecipitation assay lysis buffer. The protein concentration was measured using a BCA Protein Assay Kit (KeyGEN). Then, 30 μg of protein was separated on 10% SDS-polyacrylamide gel and transferred to polyvinylidenefluoride membrane (Merck Millipore). The membranes were blocked using western blocking buffer, then incubated with primary antibodies (TRIM29, ab108627; KRT5, ab8068; GAPDH, ab8245; Abcam) overnight at 4°C, followed by incubating with secondary antibody at 37°C for 1 h. The bands were visualised using ECL detection kit (P0018S, Beyotime). GAPDH was the normalisation.

For the determination of protein stability, the cells were treated with cycloheximide (CHX) to inhibit protein synthesis. The protein levels of KRT5 in CHX treated cells for 0, 2, 4, 8 h were detected by western blot. According to the signal intensity of protein bands, the relative expression of each protein was calculated and the half-life curve of KRT5 protein was drawn.

After transfection of oe-TRIM29, SW480 and HCT116 cells were treated by MG132 (20 μM) for 2 h to block proteasome degradation pathway. Then, the protein levels of KRT5 were detected.

### 2.8 CCK-8 method

A 96-well plate was equipped with 100 μL cell suspension at a density of 2 × 10^4^ cells/mL. The plates were precultured in an incubator at 37°C under 5% CO_2_ for 24 h. The CCK-8 solution (10 μL, AMJ-KT0001, AmyJet Technology, China) was added to each well, and the cells were incubated for 1 h. The absorbance was measured at 450 nm using a microplate reader (HBS-1096A, Nanjing DeTie Experimental Equipment Co., Ltd, China).

### 2.9 Colony formation assay

The SW480 and HCT116 cells were inoculated into six-well plates and cultured for 14 days with the culture medium refreshing every 2 days. Thereafter, cells were stained by crystal violet (0.1%) for 10 min. Colonies have been observed by a microscope (Nikon, Tokyo, Japan).

### 2.10 EdU assay

A BeyoClick™ EdU Imaging Detection Kit (C0071S, Beyotime) was used to detect cell proliferation. SW480 and HCT116 cells were fixed using 4% paraformaldehyde (PFA, 47608, Sigma-Aldrich, St. Louis, MO, USA), permeabilised using Triton X-100 (93443, Sigma-Aldrich), and then incubated with the Click-iT EdU reaction cocktail for 30 min. 4,6-diamidino-2-phenyl indole was used to stain the DNA and visualise cell nuclei. The stained cells were photographed using a fluorescence microscope (Olympus, Tokyo, Japan).

### 2.11 Co-immunoprecipitation (CO-IP)

The cell lysate containing the protease inhibitor was added to the collected cells, and the cells were lysed at 4°C for 30 min. After centrifugation, protein concentration was measured using the BCA method. The supernatant was denatured and used as the input. Following this, 1.0 µg IgG and 20 µL protein A/G beads were added into the IgG group and 20 µL protein A/G beads were added to the IP group and incubated at 4°C for 1 h. After centrifugation, the supernatant was collected and incubated at 4°C overnight after addition of the antibody, and 80 µL protein A/G-beads were added to the cells and the cells were incubated at 4°C for 2 h. The immunoprecipitated complexes were collected via centrifugation and washed four times with 1 mL of precooled lysate (without inhibitor). Supernatants were collected after centrifugation. Finally, 80 µL of 1× reduced loading buffer was added and boiled in boiling water for 10 min, and 10 µL of the supernatant was collected for western blot detection after centrifugation.

### 2.12 Data analysis

All data were collected from at least three independent experiments and analysed using the GraphPad Prism software (version 7.0). Comparisons between two groups or among multiple groups were assessed using Student’s *t*-test or one-way ANOVA. Data are presented as the mean ± SD. Statistical significance was set at *P* < 0.05.

## 3 Results

### 3.1 Overexpression of TRIM29 in colon cancer

**Figure 1:**
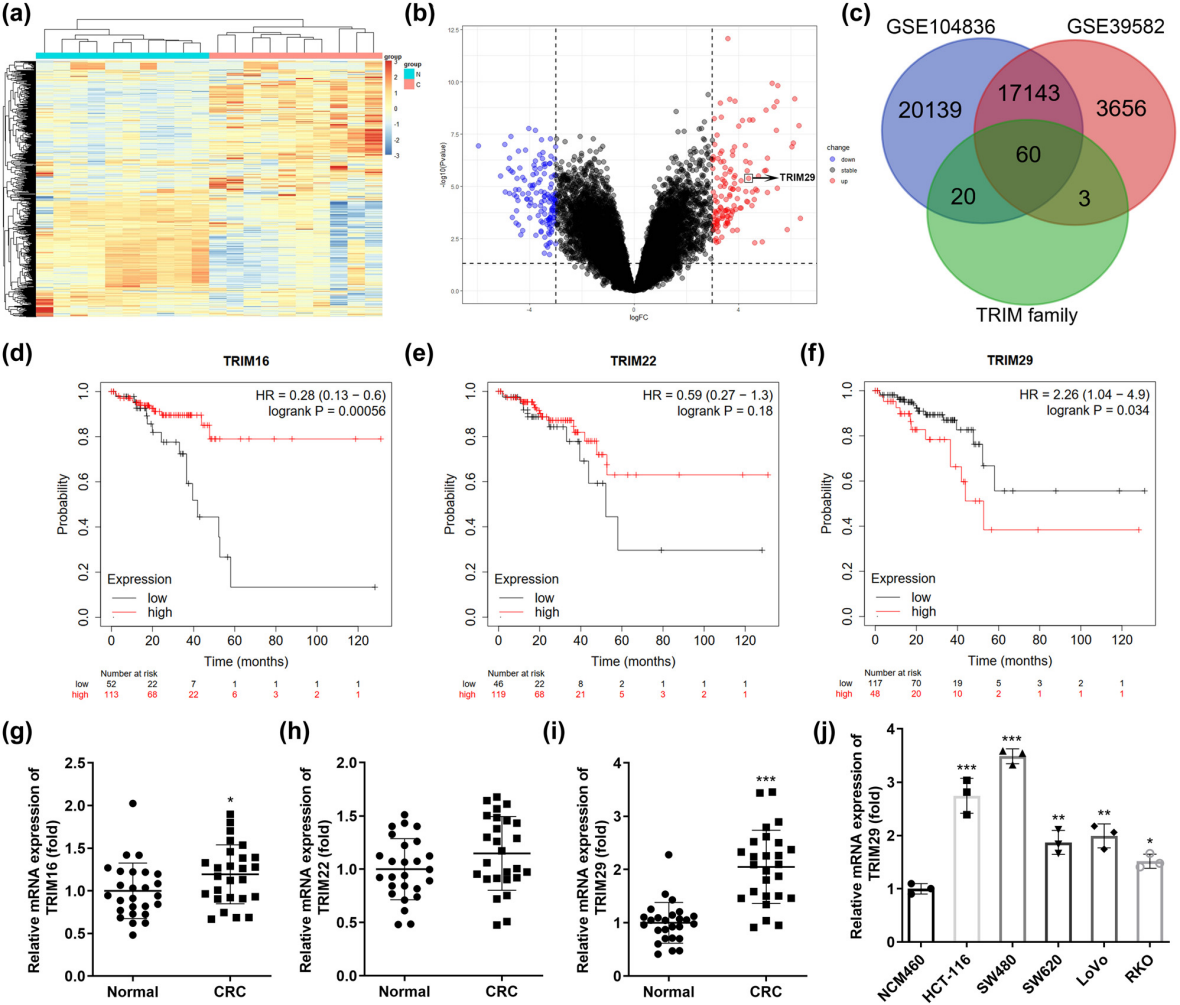
TRIM29 upregulation in colon cancer. Heatmaps (a) and volcano maps (b) of differently expressed genes in colon cancer obtained from the GSE10483 database. (c) Differentially expressed genes belonging to the TRIM family in GSE104836 and GSE39582 databases are shown as Venn diagrams. Using Kmplot software, the survival curves of TRIM16 (d), TRIM22 (e), and TRIM29 (f) in patients with colon cancer were obtained. TRIM16 (g), TRIM22 (h), and TRIM29 (i) levels in colon cancer tissues were assessed using qPCR. (j) TRIM29 levels in colon cancer cells were assessed using qPCR. *P < 0.05, **P < 0.01, ***P < 0.001 vs Normal group or NCM460.

Using bioinformatics analysis, upregulated and downregulated genes were identified and expressed as heatmaps (Figure 1a) and volcano maps (Figure 1b). TRIM29 expression was upregulated in colon cancer. Differentially expressed genes belonging to the TRIM family in GSE104836 and GSE39582 databases are expressed as Venn diagrams (Figure 1c). The differential expression levels of TRIM16 (Figure 1d), TRIM22 (Figure 1e), and TRIM29 (Figure 1f) were significant. The survival curves of TRIM16, TRIM22, and TRIM29 in colon cancer patients were obtained using Kmplot software (https://kmplot. com/analysis/). Patients with low levels of TRIM16 or high levels or TRIM29 levels had a poor prognosis. Furthermore, compared with normal controls, TRIM16 (Figure 1g) and TRIM29 (Figure 1i) were significantly upregulated in colon cancer tissues whereas TRIM22 (Figure 1h) showed no difference. The difference in TRIM29 expression was the most significant. Therefore, TRIM29 was selected for subsequent experiments. Moreover, compared to NCM460 cells, TRIM29 was significantly upregulated in colon cancer cells, especially in SW480 and HCT-116 cells (Figure 1j).

### 3.2 TRIM29 silencing prevents the growth of colon cancer cells

**Figure 2:**
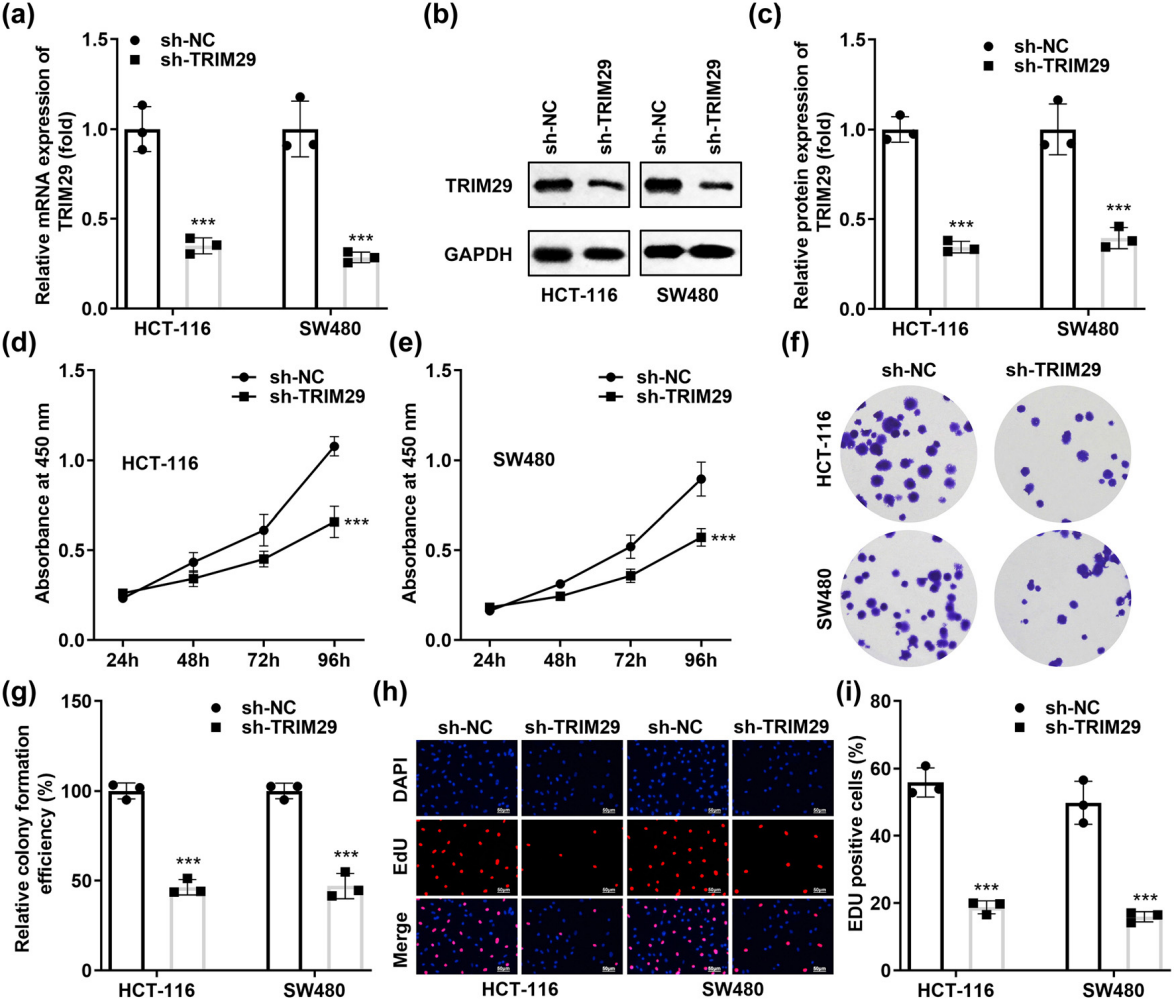
TRIM29 silencing prevents the growth of colon cancer cells. The transfection efficiency of sh-TRIM29 1# and 2# was tested using qPCR (a) and western blotting (b) and (c). Cell proliferation in colon cancer cells transfected with sh-TRIM29 1# and 2# was detected using CCK-8 (d) and (e), colony formation (f) and (g), and EDU assays (h) and (i). ***P < 0.001 vs sh-NC.

For TRIM29 knockdown, sh-TRIM29 1# and 2# were transfected into SW480 and HCT-116 cells. The transfection efficiency was tested using qPCR (Figure 2a) and western blotting (Figure 2b and c). Our results showed that the mRNA and protein levels of TRIM29 significantly decreased after sh-TRIM29 1# and 2# transfection. After sh-TRIM29 1# and 2# transfection, the cell viability (Figure 2d and e), colony formation (Figure 2f and g), and number of EDU positive cells (Figure 2h and i) significantly declined, suggesting that TRIM29 silencing decreased the proliferation of colon cancer cells.

### 3.3 TRIM29 silencing enhances KRT5 levels by decreasing ubiquitination of KRT5

**Figure 3:**
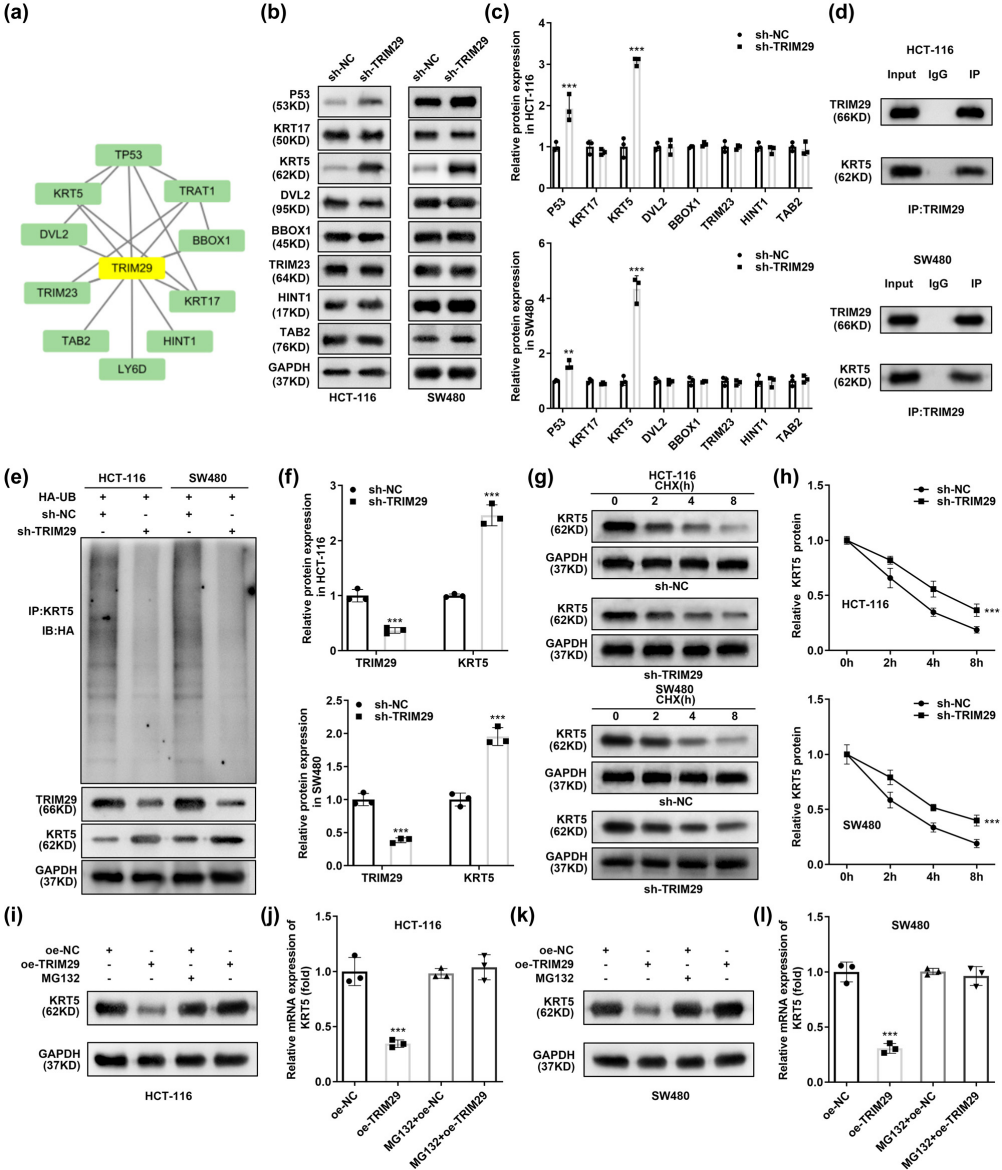
TRIM29 silencing enhances KRT5 levels by declining ubiquitination of KRT5. (a) STRING online database analysis was used to select TRIM29 related genes. (b) and (c) Following sh-TRIM29 transfection, the protein levels of genes related to TRIM29 were determined using western blotting. (d) Co-IP assay was performed to detect the interaction between TRIM29 and KRT5. (e) and (f) Ubiquitination levels of KRT5 and protein levels of TRIM29 and KRT5 in colon cancer cells transfected with sh-TRIM29 were assessed using western blotting. (g) Protein levels of KRT5 at different time points in colon cancer cells transfected with CHX and sh-TRIM29. (h) Quantitative results of protein levels of KRT5 at different time points in colon cancer cells treated with CHX and sh-TRIM29. (i–l) After transfection of oe-TRIM29, HCT-116 and SW480 cells were treated with MG132. Then the expression of KRT5 was evaluated by western blot. ***P < 0.001 vs sh-NC.

Using STRING database analysis, we identified ten genes closely related to TRIM29 (Figure 3a). Western blot analysis revealed that after TRIM29 knockdown, only P53 and KRT5 were significantly upregulated (Figure 3b and c). No study has reported the role of TRIM29 and KRT in colon cancer. KRT5 cells were selected for subsequent experiments. The relationship between TRIM29 and KRT5 was further examined using a CO-IP assay, which confirmed that TRIM29 and KRT5 can be combined (Figure 3d). Besides, in SW480 and HCT-116 cells, ubiquitination of KRT5 was downregulated by TRIM29 knockdown. TRIM29 protein levels were decreased by TRIM29 knockdown whereas KRT5 protein levels were enhanced by TRIM29 knockdown (Figure 3e and f). Furthermore, CHX-induced degradation of KRT5 in SW480 and HCT-116 cells was reversed by TRIM29 knockdown (Figure 3g and h). In addition, we treated SW480 and HCT-116 cells with MG132 to block the proteasome degradation pathway. Without MG132, TRIM29 overexpression notably reduced the level of KRT5. However, after treatment of MG132, this inhibitory effect of TRIM29 disappeared (Figure 3i–l). These results imply that TRIM29 silencing enhanced KRT5 levels by decreasing the ubiquitination of KRT5 and elevating that stability of KRT5 protein.

### 3.4 KRT5 overexpression prevents the growth of colon cancer cells

**Figure 4:**
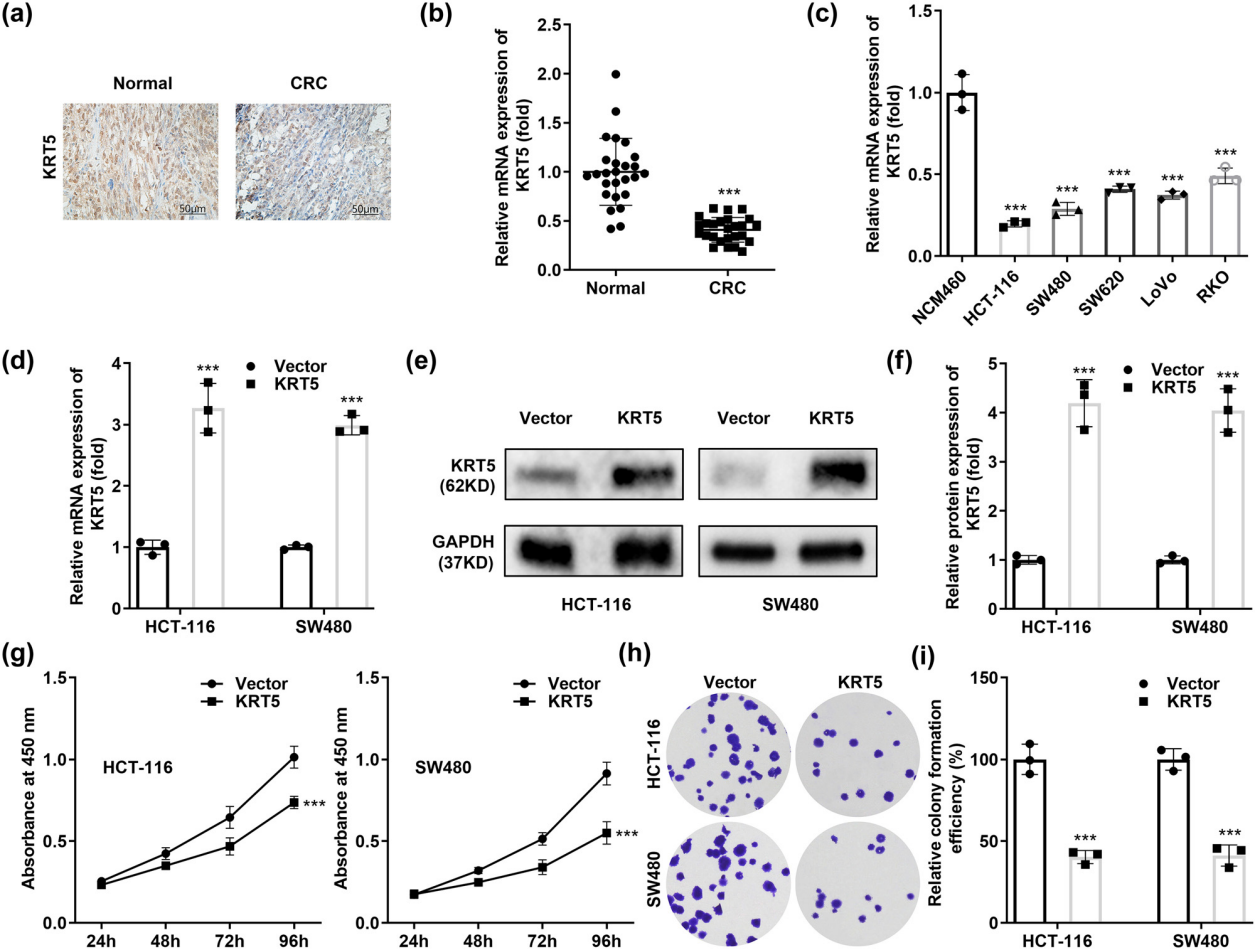
KRT5 overexpression prevents the cell growth of colon cancer cells. (a) and (b) KRT5 levels in colon cancer tissues were detected via immunohistochemistry and qPCR. (c) KRT5 levels in colon cancer cells were detected using qPCR. The transfection efficiency of KRT5 was tested using qPCR (d) and western blotting (e) and (f). The cell proliferation in colon cancer cells transfected with KRT5 was detected with CCK-8 (g) and colony formation (h) and (i). ***P < 0.001 vs Vector.

Next, we explored KRT5 levels in colon cancer tissues. Using immunohistochemistry (Figure 4a) and PCR (Figure 4b), we found that KRT5 expression was significantly decreased in colon cancer tissues. Similarly, compared to that in NCM460 cells, KRT5 was significantly downregulated in colon cancer cells, especially in SW480 and HCT-116 cells (Figure 4c). After transfection with the KRT5 overexpression vector, the mRNA (Figure 4d) and protein (Figure 4e and f) levels of KRT5 significantly increased. After KRT5 overexpression, the cell viability (Figure 4g) and colony formation (Figure 4h and i) of colon cancer cells significantly decreased.

### 3.5 KRT5 silencing neutralises the role of sh-TRIM29 in colon cancer cells

**Figure 5:**
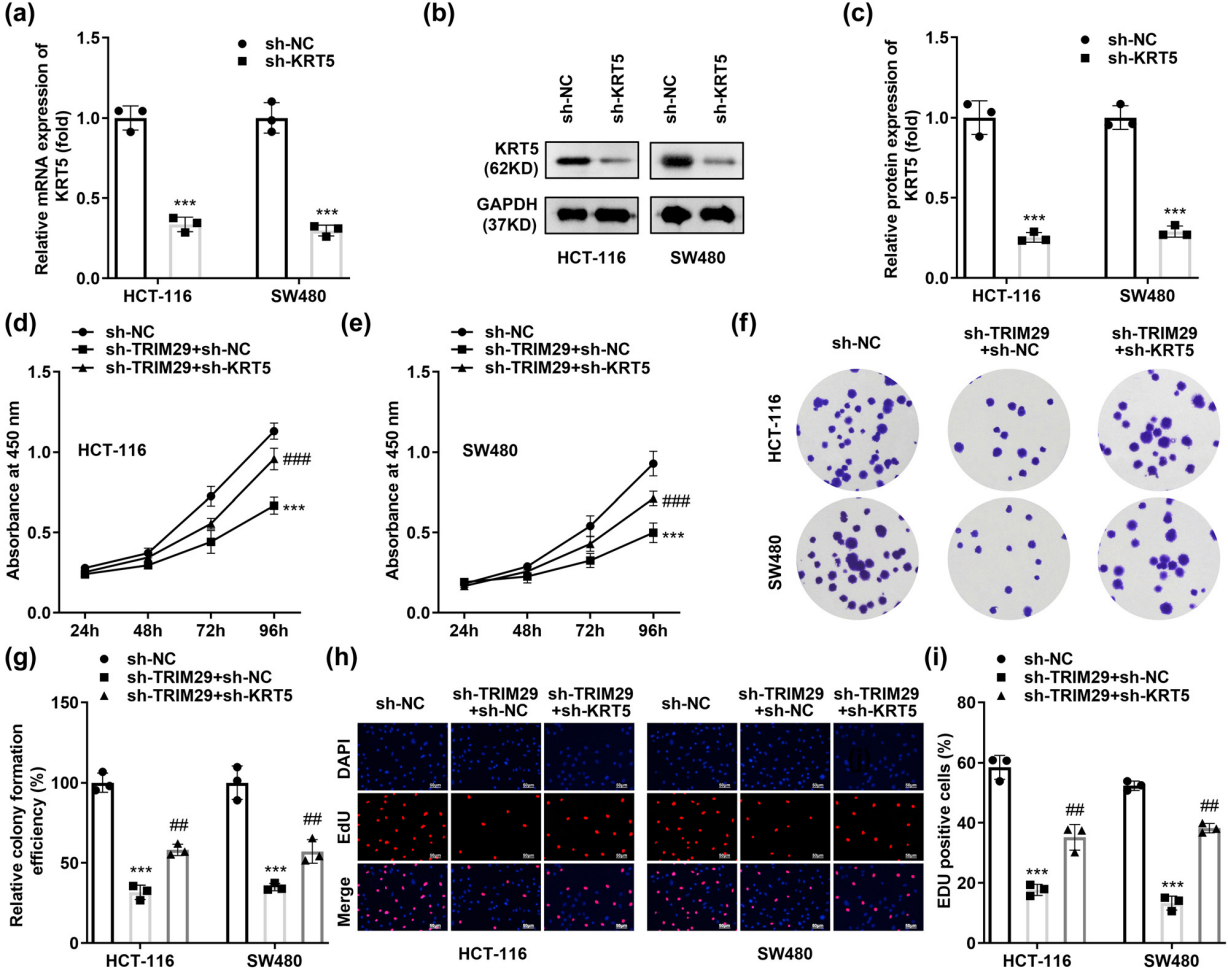
KRT5 silencing neutralises the role of sh-TRIM29 in colon cancer cells. The transfection efficiency of sh-KRT5 was tested using qPCR (a) and western blotting (a–c). Cell proliferation in colon cancer cells transfected with sh-TRIM29 and sh-KRT5 was detected using CCK-8 (d) and (e), colony formation (f) and (g), and EDU assays (h) and (i). ***P < 0.001 vs sh-NC. ##*P* < 0.01 vs sh-TRIM29 + sh-NC, ###*P* < 0.001 vs sh-TRIM29 + sh-NC.

For KRT5 knockdown, sh-KRT5 was transfected into SW480 and HCT-116 cells. The transfection efficiency was tested with qPCR (Figure 5a) and western blotting (Figure 5b and c). The mRNA and protein levels of KRT5 significantly decreased after sh-KRT5 transfection and the cell viability (Figure 5d and e), colony formation (Figure 5f and g), and number of EDU positive cells (Figure 5h and i) in sh-TRIM29-treated SW480 and HCT-116 cells significantly increased, suggesting that KRT5 silencing neutralised the role of sh-TRIM29 in colon cancer cells.

## 4 Discussion

In the present study, we explored the specific mechanism of action of TRIM29 in colon cancer progression. TRIM29 knockdown inhibited the proliferation of colon cancer cells by downregulating KRT5 ubiquitination

TRIM family proteins play an important role in life activities closely related to the occurrence and development of tumours, apoptosis, and viral response [24,25]. In this study, we evaluated the expression of TRIM16, TRIM22, and TRIM29 in colon cancer. TRIM16 and TRIM29 was upregulated in colon cancer. However, the bioinformatics analysis indicated that TRIM16 was co-related with good prognosis of colon cancer. This is strange and we cannot explain this result. Thus, we chose TRIM29 for the further study.

TRIM29, a member of the TRIM family, is located on human chromosome 11q23. Unlike other Trim family members, TRIM29 lacks a RING-finger domain. Previous studies have shown that TRIM29 participates in the occurrence and development of a variety of tumours by regulating biological processes such as cell proliferation, apoptosis, invasion, and migration [26]. However, the specific role of TRIM29 may differ in different tumours. For example, TRIM29 is upregulated in colorectal cancer, and other tumours [13,27,28] whereas it is downregulated in breast cancer, and other tumours [29,30]. These results suggest that TRIM29 simultaneously functions as a tumour suppressor gene and an oncogene. However, studies on the role of TRIM29 in colon cancer are limited. Lei et al. [31] found that TRIM29 elevated the sensitivity of colon cancer cells to oxaliplatin by preventing transcription of KRT5. The present study demonstrated that TRIM29 was upregulated and acted as an oncogene in colon cancer. TRIM29 knockdown effectively prevented the growth of colon cancer cells, indicating its potential role in colon cancer.

Ubiquitination, a post-translational modification of proteins, plays a role in many intracellular reactions such as the cell cycle and apoptosis [32]. Abnormal ubiquitination alters the regulation of intracellular physiological activities, which may lead to cancer [33]. Protein degradation via the ubiquitin-proteasome pathway is an important function of ubiquitination [34].

As a ubiquitin E3 ligase, TRIM29 has been reported to regulate the ubiquitination of proteins. For instance, TRIM29 negatively controls antiviral immune response through targeting STING for degradation [35]. TRIM29 silencing attenuates cancer stem cell-like characteristics of pancreatic ductal adenocarcinomas via regulating ISG15 ubiquitination and degradation [16].

As a tumour suppressor, KRT5 participates in the expression and regulation of cell cycle progression and DNA damage repair in normal cells. In bladder cancer, KRT5/6 and KRT20 are used as combined markers in immunohistochemical analysis, which is helpful for the clinical evaluation of patient benefits from chemotherapy [36]. In SK-OV-3 cells, KRT5 knockdown prevents cell migration [37]. Furthermore, mutation of KRT5 in the muscle cells of mice with squamous differentiation leads to a higher incidence of invasive bladder cancer [38]. With continuous research, KRT5 has been reported to be regulated by many different mechanisms. Du et al. [39] demonstrated that microRNA-601 silencing prevents the growth and metastasis of prostate cancer stem cells by enhancing KRT5 expression. Zhang et al. [40] confirmed that forkhead box M1 enhances the migratory ability of SK-OV-3 cells by promoting KRT5 expression through binding to a consensus AP-2 cis-element. Thus, the effects of KRT5 on cancer are regulated by multiple mechanisms.

However, the association between TRIM29 and KRT5 in colon cancer remains unclear. In this study, we confirmed that TRIM29 and KRT5 can be combined using a CO-IP assay. Additionally, TRIM29 knockdown decreased the ubiquitination of KRT5 and prevented degradation of KRT5. KRT5 silencing neutralises the inhibitory effects of sh-TRIM29 on colon cancer cell growth.

In conclusion, our study demonstrated that TRIM29 was upregulated in colon cancer and TRIM29 silencing inhibited colon cancer cell growth. Mechanistically, TRIM29 silencing enhanced KRT5 levels and prevented its degradation by decreasing the ubiquitination levels. However, this study had some limitations. Owing to limited conditions, our study lacks validation by *in vivo* experiments and clinical studies and future studies should focus on these aspects. In future, we will conduct additional clinical studies and *in vivo* experiments to further explore the role of TRIM29/KRT5 axis in colon cancer progression.
